# P04.90. Review of pharmacoeconomic evaluations on Kampo medicine in Japan

**DOI:** 10.1186/1472-6882-12-S1-P360

**Published:** 2012-06-12

**Authors:** W Tang, A Igarashi, K Tsutani

**Affiliations:** 1 University of Tokyo, Tokyo, Japan

## Purpose

Kampo (Japanese traditional herbal medicine) plays a certain role in the current health practices in Japan. Similar to other medical interventions, health-economic aspects of Kampo medicines should be assessed for the rational use of scarce healthcare resources. The purpose of this study is to review the published pharmacoeconomic evaluations on Kampo medicines in Japan, delineating the current situation in this area.

## Methods

We searched the database “Ichushi (Japana Centra Revuo Medicina) Web (Ver.5)” which is the largest database of medical literature in Japan, using search terms of “Kampo” and “keizai” (economics). Literature published between 1983 and 2011 were eligible for searching. After primary screening, we selected articles regarded as full economic evaluations or cost studies. Structured abstracts designed especially for health-economic analyses of Kampo medicines were composed for them.

## Results

Although we found 110 articles via initial electric search, only 11 articles on Kampo medicines (10 full economic evaluations and 1 cost analysis) were eligible for next phase, i.e., composing a structured abstract. The most common design of full economic evaluations is cost-consequence analysis (CCA) (n=8), which did not aggregate costs and consequences in the conclusions. However, considering the measurement of consequences adopted in those CCAs, 6 evaluations were similar to cost-effectiveness analysis (CEA) and the other 2 were similar to cost-minimization analysis (CMA). No cost-utility analyses (CUA) were found in our review.

## Conclusion

The published pharmacoeconomic evaluations on Kampo medicines in Japan are few and the quality of them needs to be improved. The spread of essential pharmacoeconomic knowledge among researchers is considered to be important for the future economic evaluations on Kampo medicines and its use in health policy and practices in Japan.

